# Modulation of Social Cognition *via* Hallucinogens and “Entactogens”

**DOI:** 10.3389/fpsyt.2019.00881

**Published:** 2019-12-03

**Authors:** Katrin H. Preller, Franz X. Vollenweider

**Affiliations:** Neuropsychopharmacology and Brain Imaging, Department of Psychiatry, Psychotherapy, and Psychosomatics, University Hospital for Psychiatry Zurich, Zurich, Switzerland

**Keywords:** social cognition, psychedelics, serotonin, pharmacology, functional magnetic resonance imaging, emotions

## Abstract

Social cognition is a fundamental ability in human everyday lives. Deficits in social functioning also represent a core aspect of many psychiatric disorders. Yet, despite its significance, deficits in social cognition skills are insufficiently targeted by current treatments. Hallucinogens and entactogens have been shown to have the potential to modulate social processing. This article reviews the literature on the influence of hallucinogens and entactogens on social processing in controlled experimental studies in humans and elucidates the underlying neurobiological and neuropharmacological mechanisms. Furthermore, it identifies current knowledge gaps and derives implications for hallucinogen-assisted treatment approaches as well as the development of novel medication for trans-diagnostic impairments in social cognition.

## Introduction

Humans are a social species (1). Social processes range from societal matters like politics, to more private every-day activities like being successful in a working environment, finding an apartment, romantic partnerships, and the use of virtual social networks. To be able to function in this social environment, we use capabilities which are subsumed under the term “social cognition” (2). Social cognition has been defined as mental processes through which we perceive, think about, and act toward other people (3). Critically, deficits in social functioning represent a core aspect and important diagnostic criterion of many—if not all—psychiatric disorders (4). Not only do difficulties in social interaction abilities increase the risk of developing a psychiatric disorder, but they also contribute to the maintenance or worsening of symptoms, as therapeutic processes as well as support seeking and re-integration into everyday activities, e.g., work-life, are social activities (4, 5). The importance of social cognition has also been recognized by the Research Domain Criteria (RDoC) initiative, which defines social processes as one of five trans-diagnostic dimensional constructs critical to human behavior and mental disorders (6–8). Yet, deficits in social cognition skills are insufficiently—if at all—targeted by current treatment approaches (9).

Hallucinogens are psychoactive substance which induce transient perceptual anomalies and an altered state of consciousness. The effect of entactogens is characterized by experiences of oneness and emotional openness. Entactogens as well as hallucinogens have been shown to successfully modulate social processing in rigorous scientific studies (10–12). This is important for two reasons (13): 1) In the search for novel medication for transdiagnostic social dysfunction in psychiatric disorders, these substances provide a powerful tool to increase our understanding of the neural mechanisms underlying social processing and behavior. Due to their well-investigated receptor pharmacology, in particular with regard to hallucinogens, they can identify novel targets for the development of new therapeutics. 2) Given that drug development in psychiatry has stagnated for decades, new therapeutic models are urgently needed (14). Entactogens and hallucinogens have shown promising results in preliminary clinical trials in disorders also characterized by social impairments such as depression, anxiety, post-traumatic stress disorder (PTSD), and autism spectrum disorders (ASD) (15–19). These substances could, therefore, represent important adjuncts to psychotherapy in psychiatric disorders.

The first part of this review focuses on the effects of hallucinogens and entactogens on social cognition in clinical populations (*Modulation of Social Cognition in Clinical Populations*). *Acute Effects of Entactogens and Hallucinogens on Social Cognition in Healthy Volunteers* reviews the acute effects of entactogens and hallucinogens on social cognition in healthy volunteers. *Long-Lasting Effects in Healthy Participants* provides a summary of long-lasting hallucinogen- and entactogen-induced effects on social cognition, and *Neuropharmacological Underpinnings of Alterations in Social Cognition Induced by Hallucinogens and Entactogens* explores the neuropharmacological basis of these modulatory effects. This chapter is particularly important for informing the development of novel therapeutics targeting socio-cognitive deficits in psychiatric disorders. Complimentary to the work reviewed here, there is a broad body of literature on the effects of these substances on social cognition in animals (20). However, these studies are beyond the scope of this review and so will not be discussed here. Furthermore, this review focuses on experimental and controlled studies in humans and will not include literature on survey data or studies completed with recreational drug users. This review mainly discusses effects induced by two entactogens, 3,4-methylenedioxymethamphetamine (MDMA) and gamma-hydroxybutyrate (GHB), and two hallucinogens, lysergic acid diethylamide (LSD) and psilocybin. GHB, sometimes also referred to as liquid ecstasy, has been associated with the group of entactogens (21). However, it is important to note that the neuropharmacological mechanisms underlying GHB’s psychotropic effects differ strongly from MDMA and serotonergic hallucinogens (22). Yet, given that GHB has been reported to be used recreationally for its prosocial effects, empirical studies on GHB are included in this review. Experimental research on the influence of other hallucinogens and entactogens on social functioning in humans is currently lacking and should be investigated in future studies.

## Modulation of Social Cognition in Clinical Populations

Alterations in social processing may be important modulators of the clinical efficacy of entactogens and hallucinogens. MDMA-assisted psychotherapy has been shown to reduce social anxiety in autistic adults for up to 6 months after treatment (18). Twelve out of 19 PTSD patients interviewed 1 year after they had completed MDMA-assisted therapy reported enhanced relationships and social functioning as a benefit from participating in the treatment (23). The patients described increased empathy, communication with other people, and improved relationships with friends and family (23). These pro-social effects may be particularly important for preventing relapse and increasing the long-term success of MDMA-assisted therapy, since they may reduce social withdrawal and promote support seeking.

Recent preliminary studies on the efficacy of psilocybin in mood disorders and addiction have also shown promising results (16, 17, 24, 25). In an open-label pilot study, 12 out of 15 treatment-seeking smokers were nicotine abstinent 6 months after two to three administrations of psilocybin (26). In a follow-up interview participants identified social factors, i.e., smoking as a way of connecting with other people, that contributed to their addiction and reported psilocybin-induced feelings of love and connection with their environment and other people as important for quitting smoking (27). Furthermore, some patients described engaging more in prosocial and altruistic activities after their psilocybin sessions (27), raising the possibility that psilocybin may have re-instated social reward processing helping patients to overcome their addiction.

Furthermore, psilocybin has been shown to have beneficial effects in an open-label feasibility study in patients suffering from treatment-resistant depression (16). Three months after treatment, patients showed increases in extraversion and openness scores (28). Furthermore, Lyons & Carhart-Harris (29) reported a slight, but non-significant decrease in authoritarian political views in seven depressed patients 7–12 months after treatment with psilocybin. In this study, objective tests of emotion recognition and processing were conducted. On the FERT, the speed of emotional face recognition was increased 1 week after psilocybin treatment, an effect that correlated with reduced anhedonia (30). In contrast to results obtained during the acute effects of psilocybin in healthy participants (see *Empathy, Mentalizing, and Emotion Recognition*), amygdala reactivity was increased in response to fearful faces in treatment-resistant depressed patients the morning after psilocybin administration (31). It is therefore possible that psilocybin facilitates the processing of negative experiences acutely *via* a reduction of amygdala reactivity, rendering them more accessible and bearable. This may lead to increased reactivity and emotional processing post-acutely. However, increased amygdala reactivity toward fearful faces was measured prior to any psychological integration work (31). Therefore, long-term effects of psilocybin on amygdala reactivity and its clinical relevance still need to be determined in future studies. Yet, the same patients reported that they experienced a sense of disconnection from others as particularly distressing before psilocybin-assisted treatment (32). After treatment, many reported to be able to “re-connect” with family members, friends, strangers, and even people who had wronged them. Patients identified this increased connection as one of two main change processes in relation to treatment (32), supporting the idea that the influence of hallucinogens on social cognition and behavior may be an important mechanism underlying their clinical efficacy.

## Acute Effects of Entactogens and Hallucinogens on Social Cognition in Healthy Volunteers

Acutely, MDMA has been described as a prototypical entactogen and is recreationally used for its prosocial effects (20). It is also the substance most widely studied in relation to social perception. More recently, there has been growing interest the effects of hallucinogens, in particular LSD and psilocybin, on social cognition. Studies showed that, like MDMA, both psilocybin and LSD, significantly modulate social processing and have acute pro-social effects (11, 12, 33, 34). Recreationally, low doses of GHB have been reported to be used to increase sexual arousal (35).

Self-reported pro-social effects measured in scientific studies include increased trust and closeness to others. For example, after the administration of 1.5 mg/kg MDMA participants reported a significantly increased desire to engage in social activities (36), as well as increased pleasantness of affective social touch (37). When given the opportunity, MDMA participants also spent more time interacting with others, particularly after a low dose (0.5 mg/kg) (38). Furthermore, MDMA (125 mg) increased the subjective experience of being close to others and trusting others by approximately 25% during peak effects (10, 39). Increases in closeness and trust during peak effects after LSD administration (200 µg) have been shown to be in a similar range (33, 40). GHB (20 mg/kg) has been reported to increase the tendency to talk (41).

**Figure 1:**
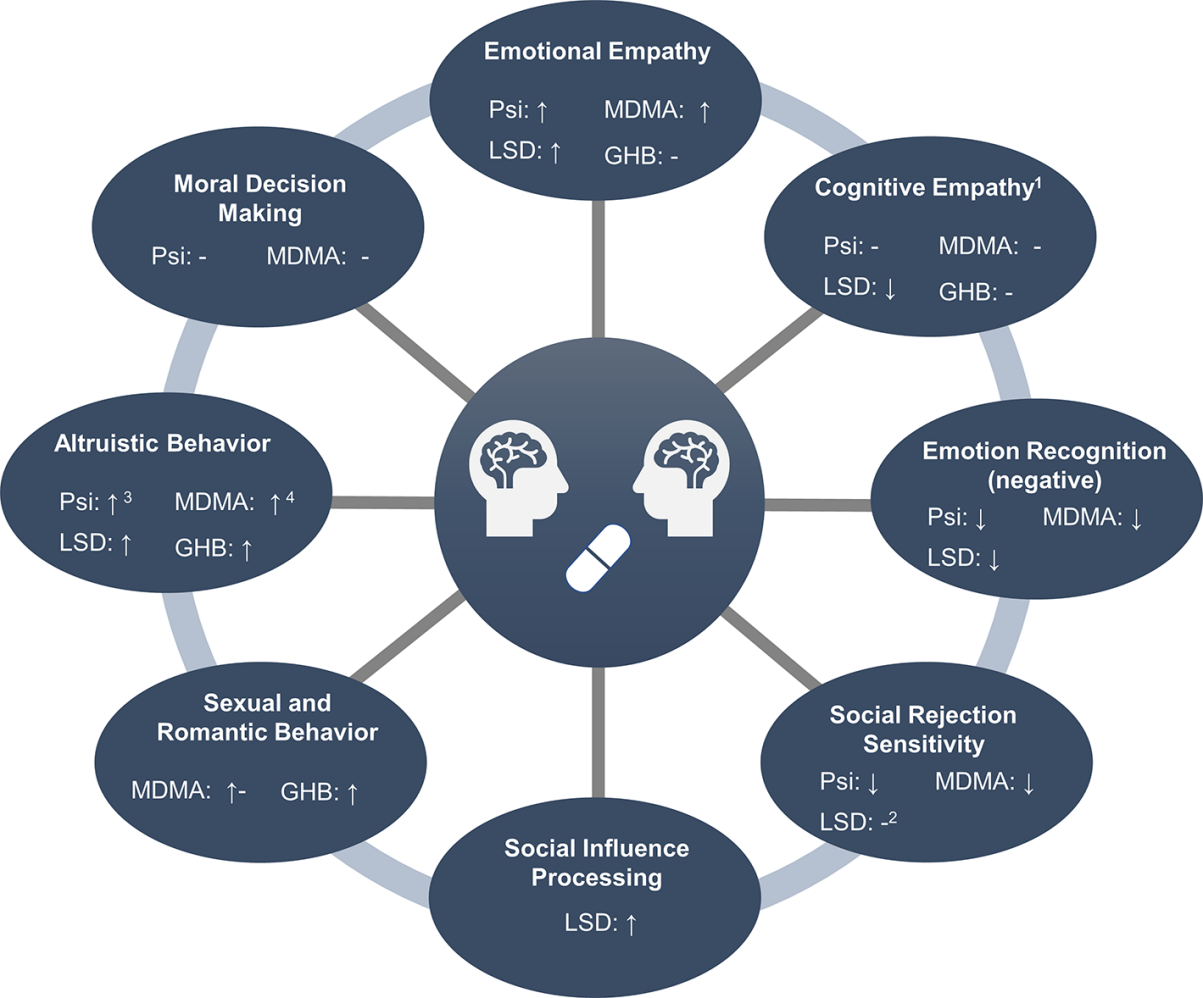
Overview of social processes modulated by entactogens and hallucinogens. ^1^Measured with the Multifaceted Empathy Test. ^2^Only assessed after the administration of low doses (≤26 µg). ^3^Assessed in male participants only. ^4^In men and when directed toward friends, but not strangers.

The following chapters provide a detailed overview of the acute effects of MDMA, GHB, psilocybin, and LSD on different objective measures of social cognition, including empathy, mentalizing, and emotion recognition, moral and altruistic behavior, social rejection sensitivity, social influence, sexual arousal and perception of romantic relationships, and social influence processing. A summary of the results is provided in **Figure 1**.

### Empathy, Mentalizing, and Emotion Recognition

Empathy has been defined as the ability to vicariously experience and/or understand the affect of others, and is thought to be critical for prosocial behavior (42). Empathy is impaired in a number of psychiatric disorders, including depression, addiction, borderline personality disorder, and psychopathy (43–46). However, empathy is a multidimensional construct, comprising of both emotional and cognitive components (43). The emotional aspect of empathy describes a person’s emotional reaction to another’s emotional state, i.e., the ability to feel what another person feels (47). Cognitive empathy refers to the ability to take another person’s perspective and the understanding of another person’s mental state, without necessarily being in the same affective state (47). Thus, cognitive empathy strongly overlaps with the concepts of affective theory of mind, mentalizing, and emotion recognition (48).

Various tasks have been applied to study empathy after the administration of hallucinogens and entactogens. The Multifaceted Empathy Task [MET (49)] captures both, emotional and cognitive empathy. Participants are asked to rate emotional pictures on induced emotional concern and arousal (emotional empathy). Furthermore, participants are asked to identify the mental state the person depicted is in (cognitive empathy). Additionally, cognitive empathy has been assessed using tasks such as the Movie for the Assessment of Social Cognition (MASC), the Reading the Mind in the Eyes Task (RMET), as well as different versions of the Facial Emotion Recognition Task (FERT). The MASC is a video-based test of mentalizing and therefore represents the most ecologically valid test of cognitive empathy (50). The REMT, like the MET, requires participants to infer the mental state of a person in a photograph by choosing which of four words provided along with the picture describes best what the person in the picture is feeling. However, while the MET displays everyday life situations conveying information on emotional mental states *via* facial expression, body language, and context, the RMET focuses exclusively on the eye region. The FERT constitutes a further emotion recognition task. In most versions, different intensities of facial emotions are presented making it possible to calculate the intensity that is necessary for an emotion to be detected correctly, but unlike the MASC and MET does not depict whole sceneries.

Various studies have consistently shown that MDMA modulates emotional empathy, assessed using the MET (49). An overview of all results is presented in **Table 1**. One hundred twenty-five milligrams as well as 75 mg MDMA increased emotional empathy (10, 51, 52). In two studies this increase was particularly pronounced in response to positive stimuli and in male participants (10, 51). A third study did not find an influence of valence (52). A pooled analyses of these data confirmed the MDMA-induced increase in emotional empathy in particular for positive emotions, but did not find an influence of sex or trait empathy (62). However, one study, showed contrary results reporting no influence of 100 mg of MDMA on emotional empathy in male participants (53).

**Table 1:** Effects of entactogens and hallucinogens on empathy, mentalizing, and emotion recognition.

Drug	Doses	Emotional empathy	Cognitive empathy			Emotion recognition		References
		MET	MET	MASC	RMET	FERT/Affective Bias Task	Emotional face reactivity	
MDMA	75 mg	↑**^1^**	**–**	**–**	**–**	**–**		51, 52
MDMA	100 mg	**–**	**–**			↓**^2^**		53
MDMA	125 mg	↑**^1^**	**–**		↑↓**^3^**	↓**^2^**		10, 39, 54
MDMA	0.75 mg/kg				**–**	**–**	**–**	36, 37, 55, 56
MDMA	1.5 mg/kg				**–**	↓**^2^**	↑**^4^**	36, 37, 55, 56
GHB	20 mg/kg	**–**	**–**	**-–**				41
Psilocybin	0.115 mg/kg						↓**^5^**	57
Psilocybin	0.160 mg/kg						↓**^6^**	58
Psilocybin	0.170 mg/kg						↓**^7^**	59
Psilocybin	0.215 mg/kg	↑	**–**		↓**^2^**			12, 60
LSD	100 μg	**–**	↓			↓**^2^**	↓**^8^**	33, 61
LSD	200 μg	↑**^9^**	↓			↓**^2^**		33

^1^Predominantly in male participants and for positive stimuli.

^2^For negative emotions.

^3^Decreases for negative emotions, increases for positive emotions.

^4^Increased zygomatic (“smile”) muscle activity in response to happy facial expressions, increased visual attention to happy faces.

^5^Reduced response to negative emotions (EEG).

^6^Connectivity changes during negative and positive facial emotion processing (fMRI).

^7^Reduced neural response to negative and positive facial emotions (EEG).

^8^Reduced neural response to negative emotions (fMRI).

^9^For positive stimuliMET, Multifaceted Empathy Test; MASC, Movie for the Assessment of Social Cognition; FERT, Facial Emotion Recognition Task; empty cells indicate that this measure was not collected.

Like MDMA, LSD has been shown to increase emotional empathy, assessed with the MET. LSD dose-dependently increased emotional empathy with significant effects at 200 µg (33). In line with results obtained under the influence of MDMA, enhanced emotional empathy under LSD was not modulated by trait empathy (12). However, while the LSD-induced increase in emotional empathy was particularly pronounced for positive stimuli, the psilocybin-induced (0.215 mg/kg) increase in emotional empathy was shown to be independent of valence (12). In contrast to MDMA and LSD, GHB (20 mg/kg) was not shown to affect emotional empathy (41).

While the increase in emotional empathy after the administration of MDMA, psilocybin, and LSD is mostly consistent across studies and substances, their effect on cognitive empathy is less clear. Performance on the MASC has been studied after the administration of GHB (20 mg/kg), but did not reveal significant results (41). Similarly, MDMA (75 mg) administration did not induce significant modulations on the MASC (61). In line with this, MDMA (75, 100, & 125 mg), GHB (20 mg/kg), and psilocybin (0.215 mg/kg) did not affect cognitive empathy on the MET (10, 12, 51–53, 62). In contrast, LSD (100 and 200 µg) decreased cognitive empathy on the MET (33). In line with results obtained with the MET, two studies did not find an effect of MDMA (0.75 mg/kg, 1.5 mg/kg, and 75 mg) on the RMET (52, 55). However, a third study reported that MDMA (125 mg) increased the recognition of positive and decreased the identification of negative emotions (54). Psilocybin (0.215 mg/kg) decreased the recognition of negative emotions on the RMET (60).

Testing the performance on the FERT, Schmid et al. (57) did not find any effects after the administration of 75 mg MDMA. However, a 100 mg dose of MDMA decreased the accuracy of identifying fear and anger on a similar task (53). Additionally, a 125 mg dose of MDMA impaired the identification of fearful faces (39) and of fearful, angry, and sad faces, particularly in women, in a second study (10). This is in line with further results showing that 1.5 mg/kg but not 0.75 mg/kg MDMA decreased the accuracy of fear recognition (55) and anger and fear recognition (36). Furthermore, Wardle and de Wit (56) reported that MDMA (1.5 mg/kg) increased the intensity required to identify anger. No effects were found for a lower dose (0.75 mg/kg) or other emotions including fearful facial expressions. In the same study, MDMA (1.5 mg/kg) reduced corrugator (“frown”) muscle activity to happy facial expressions in female participants and increased zygomatic (“smile”) muscle activity to happy facial expressions in all participants. No effects were found while viewing negative emotions (56). When presented with pairs of faces (one neutral face and one emotional expression face) 1.5 mg/kg, but not 0.75 mg/kg MDMA, increased visual attention to happy faces, but not to negative emotions (37).

Emotion recognition has also been investigated after the administration of hallucinogens. Emotional face identification was not altered by small doses (“microdoses,” 6.5, 13, and 26 µg) of LSD (63). However, psychedelic doses of LSD (100 and 200 µg) impaired the recognition of fearful and sad faces on the FERT (33). In line with this, the administration of LSD (100 µg) reduced the neural response to fearful vs. neutral faces in the left amygdala and the right medial frontal cortex (61). Psilocybin (0.115 mg/kg) also reduced the subjective discrimination between fearful and neutral faces and the encoding of fearful faces measured with EEG expressed by reduced N170 responses (57). The processing of happy faces was not affected (57). However, after the administration of 0.170 mg/kg two time periods of psilocybin-induced modulation of emotional face processing were identified: during the 168–189 ms interval decreased activity in response to both neutral and fearful faces within limbic areas, including amygdala and parahippocampal gyrus, and the right temporal cortex was observed, and over the 211–242 ms interval reduced activity in response to happy faces within limbic and right temporo-occipital brain areas was observed (59). Investigating the effect of psilocybin (0.160 mg/kg) during the discrimination of angry, happy, and fearful *vs.* neutral faces on amygdala seed-to-voxel connectivity *via* functional magnetic resonance imaging (fMRI) showed that psilocybin decreased the connectivity between the amygdala and the striatum during angry face discrimination. The connectivity between the amygdala and the frontal pole was decreased during happy face discrimination. No effect was observed during discrimination of fearful faces (58).

In sum, both hallucinogens and the entactogen MDMA, but not GHB, have been shown to acutely increase emotional empathy in controlled experimental trials. This effect seems to be more pronounced for positive emotions, in particular after the administration of MDMA. Cognitive empathy and mentalizing, i.e., the ability to correctly infer another person’s mental state, was mostly unchanged by hallucinogens and entactogens. Reduced emotional but preserved cognitive empathy has been reported in patients suffering from substance use disorders (46). Facilitating the reconnection with their social environment *via* increased emotional empathy may therefore contribute to clinical efficacy of hallucinogens shown in preliminary studies with addicted patients (24, 26). In contrast to psilocybin and MDMA, LSD decreased the correct interpretation of ecologically valid stimuli (33) and psilocybin decreased the ability to infer negative emotions from the eye region (60). In one study, MDMA decreased the decoding of negative emotions from the eye region, while at the same time increasing this ability for positive stimuli (54). These results are in line with reduced recognition and processing of predominantly negative emotional faces after the administration of higher doses of MDMA and at all doses tested (low-high) of psilocybin and LSD. The increased empathy for positive emotions and decreased recognition of negative emotions shown in these studies is in line with the interpretation by Bedi et al. (55) that a decreased ability to identify negative emotions might facilitate social approach behavior and thus social interaction. This effect might be clinically relevant, since it may reduce social withdrawal behavior and improve the patient-therapist relationship during hallucinogen-assisted treatment. However, this hypothesis remains to be tested by future studies.

### Moral and Altruistic Behavior

Moral and altruistic behaviors are fundamental for a functioning society (64). Despite its significance, to date the neuropharmacology of moral behavior has been scarcely investigated. Using moral dilemma tasks, it has been shown that neither MDMA (75 mg) nor psilocybin (0.215 mg/kg) influenced moral decision making (12, 51). However, no other studies have investigated the influence of hallucinogens or entactogens on moral behavior.

To understand the effects of hallucinogens and entactogens on altruistic behavior, most studies implemented resource allocation tasks. On the Social Value Orientation Test (SVO) participants act altruistically when choosing an option that maximizes the allocation for another person. While MDMA did not change behavior on the SVO when administered in a lower dose (75 mg) (51), male participants made more altruistic choices after the administration of 125 mg MDMA (10). No effect was found for female participants, potentially because they already showed high altruistic behavior following placebo administration (10). Another study employed a similar paradigm, the Welfare Trade-Off Task, and reported that participants showed more altruistic behavior after the administration of 1 mg/kg MDMA, but only if the other person was a friend, not a stranger (65). This is in line with another study showing that MDMA (75 mg) did not influence trust or reciprocity during a Trust Game played with an unknown partner (52). However, the effects of MDMA on trust and reciprocity toward a close friend were not assessed in this study. Modulation of altruistic behavior after MDMA administration therefore seems to depend on dose, gender, and social proximity. Increased altruistic behavior on the SVO was also induced by LSD (100 and 200 µg, combined groups) (33). After GHB (20 mg/kg) administration, participants also showed more altruistic behavior on the SVO and a Charity Donation Task, but only after participants who scored high at baseline were excluded from the analysis (41). No effect was found for reciprocity during a Trust Game after GHB administration (41).

Investigating allocation behavior in more reciprocal tasks, Gabay et al. (66) showed that psilocybin (2 mg, i.v.) as well as MDMA (100 mg) reduced altruistic punishment, i.e., punishment of social norm violations which are costly to the self, in the Ultimatum Game in male participants. Furthermore, Gabay et al. (53) found that male participants behave more cooperatively when interacting with trustworthy partners and show greater recovery from breaches of trust during an iterated prisoner’s dilemma after the administration of MDMA (100 mg). However, results on economic allocation games are often difficult to interpret, especially in studies investigating the effects of substances that induce altered states of consciousness. While data on reward sensitivity were collected in the MDMA condition, this was not the case for the psilocybin condition (66). It is conceivable that psilocybin may alter sensitivity to financial rewards, rendering the interpretation of allocation tasks involving monetary rewards more challenging.

In sum, hallucinogens and entactogens have been shown to increase altruistic behavior. However, it is important to bear in mind the following caveats: these substances may also alter sensitivity to financial rewards; increases in altruistic behavior may occur only in participants with low altruism at baseline; and finally increases in altruistic behavior may occur only when directed toward a friend. A summary is presented in **Table 2**. Even though the effects of hallucinogens and entactogens on prosocial behavior seem to be complex and dependent on factors such as social proximity and baseline altruism, they may be important within a therapeutic framework as increases in altruism may support reconnection with the patients’ social environment. In contrast to altruistic behavior, no effect has been found on moral decision making as measured with moral dilemma tasks. It is conceivable that hallucinogens and entactogens do not impact moral behavior, yet it is also possible that higher doses are needed to change moral decision making. Additionally, it is noteworthy that moral dilemmas often include violent and negative actions and outcomes. Yet, it has been shown that hallucinogens reduce the processing of negative stimuli. It is therefore possible that moral dilemmas were less salient after psilocybin administration. Lastly, post-acute effects of hallucinogens and entactogens have not been examined yet. It may be possible that changes in moral behavior only occur post-acutely.

**Table 2:** Effects of entactogens and hallucinogens on moral and altruistic behavior.

Drug	Doses	Moral behavior	Altruistic behavior				Reciprocity/trust		References
		Moral Dilemmas Task	Social Value Orientation Test	Welfare Trade-Off Task	Charity Donation Task	Ultimatum game	Trust game	Prisoner’s dilemma	
MDMA	75 mg	–	–				–		51, 52
MDMA	1 mg/kg			↑^1^					65
MDMA	100 mg					↑^2^		↑^2^	53, 66
MDMA	125 mg		↑^2^						10
GHB	20 mg/kg		↑^3^		↑^3^		–		41
Psilocybin	0.215 mg/kg	–							12
Psilocybin	2 mg, i.v.					↑^2^			66
LSD	100 and 200 µg								33

^1^Only toward friends, not strangers.

^2^Only in male participants.

^3^Only after participants were excluded who scored high at baseline

empty cells indicate that this measure was not collected.

### Social Rejection Sensitivity

Increased sensitivity to social rejection and exclusion is observed in many psychiatric disorders (67–69). At the same time, psychiatric patients frequently encounter social rejection (70). Normalizing increased rejection sensitivity could therefore be clinically relevant to avoid being trapped in a vicious circle that ultimately leads to social withdrawal, reduced support, and the worsening of clinical symptoms.

A commonly used paradigm to investigate the reaction to social rejection is called “Cyberball.” This paradigm consists of an interactive virtual ball-tossing game that simulates a real-life interactive experience of social exclusion (71). While in the beginning participants are usually equally involved in the game, one player is eventually excluded. In most studies, the participants themselves are the ones who are excluded and therefore rejected by the other players, which reliably induces feelings of “social pain” (72).

MDMA (0.75 and 1.5 mg/kg) has been shown to reduce the effect of social rejection on self-reported lower mood and self-esteem. The higher dose of MDMA (1.5 mg/kg) additionally increased the perceived percentage of throws received in the rejection condition (73). However, no modulatory effects of MDMA (75 mg/kg) on the reaction to social exclusion were found when only one of three players was excluding the participant (52). Social exclusion often leads to social stress (74). Yet, MDMA (0.5 and 1.0 mg/kg) did not alter the response to social stress in the Trier Social Stress Test (75).

The effect of LSD on social rejection induced by the Cyberball game has so far only been tested with very low doses (“microdoses”). Bershad et la. (63) (in press) reported that 6.5, 13, and 26 µg did not modulate the perceived number of received ball throws or influenced mood responses to rejection. Preller et al. (11) combined the Cyberball paradigm with fMRI and MRS measurements to study the effects of psilocybin on social rejection processing. After psilocybin (0.215 mg/kg) administration, participants reported a reduced feeling of social exclusion, while at the same time no significant differences were found between placebo and psilocybin with regard to perceived number of received ball throws. Furthermore, the neural response to social exclusion was decreased in the dorsal anterior cingulate cortex and the middle frontal gyrus, key regions for social pain processing. This reduction in the “social pain signal” was significantly correlated with decreased aspartate content. Furthermore, it correlated with psilocybin-induced alterations in self-processing, i.e., experience of unity (11). This is in line with a study showing that hallucinogen-induced alterations in self-processing and social cognition are intertwined (34). Such findings may be of particular interest in the treatment of psychiatric disorders characterized by an increased self-focus like depression (76). Hallucinogen-induced alterations in self-processing such as the experience of unity may reduce self-focus and concurrently improve social functioning.

In sum, psilocybin has been shown to attenuate the processing of negative stimuli which extends to negative social interaction (11, 77). While participants under the influence of psilocybin were able to correctly guess the number of received ball throws indicating that they were fully aware of being excluded, their self-reported emotional response was decreased in line with a reduction in the “social pain signal” in the anterior cingulate cortex (11). However, MDMA reduced self-reported negative effects of social exclusion, but also increased perceived ball throws, potentially indicating reduced awareness of social exclusion (73). It is therefore possible that both substances reduce the processing of social rejection *via* different mechanisms. It is also conceivable that reducing rejection sensitivity is critically involved in the potential therapeutic effects of entactogens and hallucinogens, in particular with respect to therapist–patient interaction. However, this hypothesis has not yet been tested in clinical populations. Finally, the effects of entactogens and hallucinogens on social rejection processing other than psilocybin and MDMA still need to be investigated in future studies.

### Sexual Arousal and Perception of Romantic Relationships

Engaging in romantic relationships and sexual behavior is an intimate social process and disturbances of close inter-personal relationships are prominent in psychiatric disorders (78). Yet, only a few neuropsychopharmacological studies have so far explored this aspect of social cognition. To date, no studies have experimentally investigated how hallucinogens influence sexual arousal or the perception of romantic relationships. Studies on the effects of entactogens on these processes are more common. After the administration of MDMA (125 mg), participants reported increased sexual arousal and desire (39). Furthermore, participants used more sexual and social words when discussing a close personal relationship after the administration of 1.5 mg/kg MDMA (79). On the Sexual Arousal Task, a computerized task presenting neutral as well as implicit and explicit sexual pictures, participants treated with MDMA (75 mg) sought to increase the presentation time of implicit sexual stimuli, however they did not report alterations in sexual arousal while viewing the images (80). Furthermore, no effect was found on the evaluation of romantic relationships of others (80) or on attractiveness ratings (36). Together, these results suggest only subtle, subjective effects of MDMA on sexual arousal and perception of intimate relationships, but may reflect an increased willingness to disclose personal information (79).

GHB has been reported to have pronounced effects on self-reported sexual arousal (81). In an experimental setting, GHB (20 and 35 mg/kg) dose-independently increased self-reported sexual arousal and desire (82). Furthermore, participants reported more sexual arousal while viewing erotic as well as neutral stimuli (82). Together, these results point to a prosexual effect specific for GHB. However, more research is needed to also determine the effects of hallucinogens on sexual arousal and the perception of intimate relationships.

### Social Influence Processing

Very little research has been conducted to investigate the effect of hallucinogens and entactogens on suggestibility and social influence processing, despite the fact that it is highly relevant for therapeutic interaction. So far, only two studies have been conducted, both investigating the influence of LSD on suggestibility (83, 84). The first study, which was limited by a small sample of 10 healthy volunteers, showed that LSD (40–80 µg, i.v.) enhances suggestibility on the Creative Imagination Scale, while Cued Imagery remained unaffected (83). A second study showed that LSD (100 µg, p.o) increases adaptation to opinions expressed by a norm group, but only if those opinions were not too different from the participants own (84). Furthermore, this study showed that increases in blood oxygen level-dependent signal in medial prefrontal regions were associated with altered social feedback processing. It is therefore conceivable that hallucinogens influence how participants process social feedback and how they integrate this feedback to subsequently make decisions. This finding has direct clinical relevance for therapists working with these substances within the framework of hallucinogen-assisted therapy. Furthermore, the impact of alterations in social feedback processing in a clinical setting should be evaluated and therapists trained accordingly.

## Long-Lasting Effects in Healthy Participants

While it has repeatedly been shown that hallucinogens increase the personality trait openness (85, 86), experimental studies investigating the long-term effects of entactogens and hallucinogens on social cognition and behavior remain scarce.

It has been reported that recreational MDMA users showed increased cognitive, but not emotional, empathy compared to controls on the MET and the MASC (87, 88). Furthermore, they exhibited less-self-serving behavior on a money allocation task played with a stranger (87). Interestingly, these social functions were not influenced by acute administration of MDMA in controlled studies (10, 51, 52). However, cross-sectional investigations in recreational drug users have to be interpreted with caution as they do not allow for causal inference. It is therefore possible that MDMA has post-acute positive effects on cognitive empathy and altruistic behavior, but it is also conceivable that people with high cognitive empathy and prosocial motivation are more prone to recreationally use MDMA. To test these hypotheses, the long-term effects of MDMA on social cognition in healthy individuals need to be investigated in future experimental and controlled studies.

Self-reported increases in interpersonal closeness and positive/altruistic social effects were reported 1, 2, 6, and 14 months after one and two administrations of psilocybin (89–92). Self-reported increases in positive/altruistic social effects were also shown 12 months after the administration of LSD in healthy participants (93). However, the personality trait openness was not influenced by LSD administration in this study (93).

Objective data on the long-term effects of hallucinogens on social processes is still scarce. Mason et al. (94) reported that emotional empathy on the MET was increased the morning after a psilocybin retreat. This increase was still significant after seven days, but only for negative emotions. Given the lack of further data on objective long-term effects, future studies are needed to evaluate whether hallucinogens and entactogens have a lasting impact on social processes.

## Neuropharmacological Underpinnings of Alterations in Social Cognition Induced by Hallucinogens and Entactogens

Understanding the neuropharmacological underpinnings of alterations in social cognition induced by hallucinogens and entactogens is vital to accelerate the development of novel medication for transdiagnostic social dysfunction in psychiatric disorders. MDMA, GHB, psilocybin, and LSD engage with various targets in the brain. To assess the neuropharmacological mechanisms underlying the prosocial effects of these substances, studies have investigated the neuroendocrinology after drug administration, the effects of these substances after blocking specific receptors or transporters, and have compared the effects between substances with different mechanisms of action.

MDMA interacts with numerous transporters and receptors in the brain. It releases 5-HT, NE, and, to a lower extent, DA from nerve terminals *via* action on monoamine transporters, and increases plasma levels of oxytocin, prolactin, and cortisol (10). MDMA is also a low-potency partial agonist of 5-HT receptors (95–97). GHB has direct agonist effects on GHB- and GABA_B_-receptors and neuromodulatory properties on glutamate, dopamine, serotonin, norepinephrine, and cholinergic transmission (22). Furthermore, GHB has been shown to increase plasma progesterone, but not oxytocin or testosterone levels (41).

Molecular studies have shown that the psychoactive metabolite of psilocybin, psilocin, binds to various serotonin receptors (PDSP database: https://pdsp.unc.edu/databases/kidb.php). Psilocin has high affinity and agonist activity on the 5-HT_2A_ receptor and this receptor subtype is critically implicated in psilocybin-induced effects (98). Additionally, recent evidence in humans also suggests the involvement of the 5-HT_1A_ receptor in mediating the effects of psilocybin (99). LSD has predominantly agonist activity at 5-HT2A/C, −1A/B, −6, and −7 and dopamine D2 and D1 receptors. Administering the 5-HT_2A_ receptor antagonist ketanserin before LSD administration has been shown to block LSD-induced effects, implicating activity on this receptor as vital for its effects (100).

To date, only few studies have investigated the pharmacology of MDMA-induced alterations in social cognition *via* blocking specific receptors. Kuypers et al. (52) showed that the mixed beta-adrenoreceptor blocker/5-HT_1A_ antagonist pindolol did not block MDMA-induced increases in emotional empathy. A further study showed that neither duloxetine, which inhibits MDMA-induced monoamine transporter-dependent serotonin and norepinephrine release, reboxetine, which inhibits MDMA-induced norepinephrine release, nor clonidine, which inhibits MDMA-induced transporter-independent vesicular release of norepinephrine, blocked the observed increases in decoding accuracy for positive and impaired decoding accuracy for negative stimuli on the RMET after MDMA administration (54). However, duloxetine was most effective in reducing the acute subjective MDMA effects, implicating the serotonin system as a key mechanism of action (54). This is in line with results reported by Kuypers et al. (101) showing that an MDMA-induced reduction of arousal in response to negative sounds was blocked by the 5-HT_2A_ receptor antagonist ketanserin.

The neuropeptide oxytocin has repeatedly, although not unanimously, been related to social behavior (102). Therefore, MDMA-induced increases in oxytocin levels are another candidate mechanism potentially underlying MDMA’s prosocial effects. However, so far, no study investigating the relationship between MDMA-induced effects and oxytocin plasma levels has found significant correlations with regard to social processing and behavior. For example, increases in emotional empathy, impaired identification of negative emotions, enhanced decoding of positive facial expressions, and increased altruistic choices after MDMA administration were not related to oxytocin plasma levels or other neuroendocrine effects (10, 51, 52, 54). In line with this, comparing the effects of MDMA directly with oxytocin showed differential effects of the two substances. Kuypers et al. (52) investigated the effects of MDMA and oxytocin in a within-subject design and reported that while MDMA increased emotional empathy, oxytocin did not affect measures of empathy or other social cognitive outcomes. A further study reported that, in contrast to MDMA, intranasal oxytocin enhanced recognition of negative emotional faces (36). Additionally, only modest correlations between the effects of MDMA and oxytocin in the same individuals were found. The effects of MDMA (1.5 mg/kg) on social cognition were also substantially more pronounced than the ones induced by oxytocin (40 IU) (36). Together, these results provide limited support for the hypothesis that increases in oxytocin levels underlie the prosocial effects of MDMA. This is in contrast to animal studies indicating that oxytocin plays an important role in MDMA-related social effects (103, 104). Being beyond the scope of the current review, future work should address these discrepancies between human and animal data.

Additionally, a number of studies have compared the effects of MDMA with other amphetamines as well as with modafinil. These studies indicated that MDMA has a different effect profile than other amphetamines. In contrast to MDMA, methylphenidate and modafinil increased misclassifications of emotions as angry on the FERT while MDMA increased misclassifications as happy (39). This is in line with another study showing that methylphenidate, but not MDMA, increased ratings of sexual arousal for explicit sexual stimuli (80). Neither MDMA nor methylphenidate altered appraisal of romantic relationships (80). Furthermore, methylphenidate lacked the empathy enhancing properties of MDMA (51). When compared with methamphetamine, Bershad et al. (37) showed that MDMA, but not methamphetamine, enhanced ratings of pleasantness of experienced affective touch and increased attention toward happy faces. These results indicate that the dopaminergic system, but not serotonergic neurotransmission may be involved in enhancing sexual drive, whereas the serotonin system may be involved in increasing empathy.

To illuminate the pharmacology of GHB-induced prosocial effects, one study investigated the relationship between GHB-induced alterations in outcomes on social tasks and neuroendocrine effects (41). No correlations were found between GHB-induced neuroendocrine effects and social behavior, but low progesterone levels at baseline were predictive of altruistic behavior on the SVO and a Charity Donation Task after GHB administration. This indicates that GHB induced prosocial behavior specifically in individuals with low progesterone levels (41).

Hallucinogens have been reported to exert their prosocial effects predominantly *via* agonism at the 5-HT_2A_ receptor and potentially in parallel with downstream effects on aspartate metabolism (11). Decreased recognition of negative emotions induced by psilocybin on the RMET was blocked by pretreatment with the 5-HT_2A_ receptor antagonist ketanserin (51). In line with this, LSD-induced effects on joint attention processing, self/other differentiation, and social influence processing were blocked by ketanserin (34, 84). Comparing the effects of psilocybin to those of the N-methyl-d-aspartate receptor antagonist ketamine, Schmidt et al. (57) reported differential effects, with psilocybin reducing the processing of negative faces, whereas ketamine induced an emotional blunting characterized by reduced encoding of both, negative and positive facial expressions.

Given these pharmacological results as well as the similarity of effects induced by MDMA and hallucinogens, it is conceivable that prosocial effects, in particular increased empathy and altruistic behavior, are modulated by a common mechanism, namely 5-HT_2A_ receptor stimulation. However, the functional and modulatory contribution of other receptors stimulated by these substances is scarcely investigated, in particular with regard to hallucinogens. Additional studies are needed that investigate the role of these receptor systems by selectively blocking them to comprehensively uncover the neuropharmacology underlying hallucinogen-induced modulations of social cognition. GHB which targets mainly GHB and GABA_B_ receptors has a different effect profile implicating these receptors together with the dopamine system in prosexual effects. It has to be noted that entactogens and hallucinogens are not the only psychoactive substances that modulate social cognitive functioning. For example, alcohol has been reported to facilitate social interaction (105). While this is beyond the scope of the current review, the differential effects hallucinogens, entactogens, and other psychoactive substances should be discussed in future articles to systematically increase our understanding of the pharmacology of social cognition.

## Conclusion

The current literature on experimental and controlled investigations of the influence of hallucinogens and entactogens shows that these substances are potent modulators of social cognition and behavior. While MDMA is recreationally used for its prosocial effects, this review shows that hallucinogens such as LSD and psilocybin similarly impact social cognitive measures. GHB, however, has been shown to have predominantly prosexual effects and may therefore not be classified as a typical entactogen. As described in detail in *Neuropharmacological Underpinnings of Alterations in Social Cognition Induced by Hallucinogens and Entactogens*, agonism on the 5-HT_2A_ receptor may be a common mechanism of classic hallucinogens and MDMA which underlies their prosocial effects. This is particularly important for the development of highly needed novel therapeutics targeting social deficits in psychiatric patients. Furthermore, these results implicate alterations in social processing as key mechanisms for the efficacy of psilocybin- and MDMA-assisted therapeutic approaches. In addiction disorders, the reinstatement of social reward processing may support reductions in drug intake and help overcome addiction. In anxiety and mood disorders, MDMA and hallucinogens may promote re-connection with patients’ social environment as well as support seeking and reductions in social withdrawal. Acutely, MDMA and hallucinogens may also enhance the patient-therapist relationship. Thus, it is vital that therapists working with MDMA and hallucinogens are aware of the acute effects of these substances on social cognition, including potential increases in suggestibility.

Despite recent efforts to elucidate the effects of hallucinogens and entactogens on social cognition, major knowledge gaps remain. Studies specifically investigating the dose-dependency of modulations in social cognition induced by hallucinogens and entactogens are still scarce, in particular regarding psilocybin. LSD and MDMA have shown some dose-dependent effects on empathy and altruistic behavior (10, 33, 51), indicating that robust modulations are measurable at doses of 100 mg MDMA/100µg LSD and above. Controlled studies on very low doses, so-called “microdoses,” are still rare. Bershad et al. (63) did not find an effect of doses <30 µg LSD on social rejection sensitivity and emotional face identification. Further studies investigating dose-dependency in within-subject designs are needed. Furthermore, the impact of hallucinogens other than LSD and psilocybin on social cognition has not yet been investigated. Sex-specific effects are poorly understood. It has been shown that MDMA enhances emotional empathy predominantly in male participants (10). Furthermore, altruistic behavior after psilocybin administration was only investigated in males (66). Despite their potential clinical relevance, further systematic investigations into sex-specific drug effects are lacking. Data on effects after the acute phase of substance action are often missing. The neural mechanisms underlying changes in social cognition after administration of hallucinogens and entactogens are poorly understood. Differential effects of specific receptor systems targeted by these substances need to be investigated. Objective data on social behavior within the framework of MDMA- and hallucinogen-assisted therapy are still lacking. Studies on entactogens and hallucinogens have consistently shown prosocial effects and have identified alterations in social processing and behavior as key factors for the efficacy of treatments involving these substances. Thus, investigating these questions is a promising way to increase our mechanistic, neuropharmacological understanding of social processes, advance the development of novel therapeutics, and to uncover the full potential of these substances in clinical contexts.

## Author Contributions

KP has written the manuscript. FV has revised the manuscript.

## Funding

KP and FV are financially supported by grants from the Heffter Research Institute and the Swiss Neuromatrix Foundation.

## Conflict of Interest

The authors declare that the research was conducted in the absence of any commercial or financial relationships that could be construed as a potential conflict of interest.
